# Dietary Kynurenine Pathway Metabolites—Source, Fate, and Chromatographic Determinations

**DOI:** 10.3390/ijms242216304

**Published:** 2023-11-14

**Authors:** Ilona Sadok, Katarzyna Jędruchniewicz

**Affiliations:** 1 Laboratory of Separation and Spectroscopic Method Applications, Department of Chemistry, Institute of Biological Sciences, Faculty of Medicine, The John Paul II Catholic University of Lublin, 20-708 Lublin, Poland; 2 Laboratory of Separation and Spectroscopic Method Applications, The John Paul II Catholic University of Lublin, 20-708 Lublin, Poland; jedruchniewicz.katarzyna@gmail.com

**Keywords:** tryptophan metabolites, kynurenine pathway, kynurenine, kynurenic acid, chromatographic analysis, food analysis

## Abstract

Tryptophan metabolism plays an essential role in human health. In mammals, about 95% of dietary tryptophan is metabolized through the kynurenine pathway, which is associated with the development of several pathologies, including neurodegeneration. Some of the kynurenine pathway metabolites are agonists of the aryl hydrocarbon receptor involved in metabolic functions, inflammation, and carcinogenesis. Thus, their origins, fates, and roles are of widespread interest. Except for being produced endogenously, these metabolites can originate from exogenous sources (e.g., food) and undergo absorption in the digestive tract. Recently, a special focus on exogenous sources of tryptophan metabolites was observed. This overview summarizes current knowledge about the occurrence of the kynurenine pathway metabolites (kynurenines) in food and the analytical method utilized for their determination in different food matrices. Special attention was paid to sample preparation and chromatographic analysis, which has proven to be a core technique for the detection and quantification of kynurenines. A discussion of the fate and role of dietary kynurenines has also been addressed. This review will, hopefully, guide further studies on the impact of dietary kynurenines on human health.

## 1. Introduction

Tryptophan (TRP) is an exogenous amino acid that cannot be synthesized in the human body. It must be delivered through nutritional sources and, in the organism, it is found bound to albumin or free form [1]. In the human organism, TRP is involved in the biosynthesis of protein and regulation of metabolic networks as it is a precursor for important biologically active compounds like coenzymes nicotinamide adenine dinucleotide (NAD+), and NAD phosphate (NADP+), serotonin, tryptamine, melatonin, niacin, kynurenine (KYN), and indole and indolic acid derivatives [2,3]. Its concentration in the body is lower than that of other amino acids and it might play a rate-limiting role in protein synthesis [4]. The gut microbiota utilizes ~4–6% of TRP for the production of indole, indican, tryptamine, skatole, and indole acid derivatives [5]. In the mammal’s brain, TRP is metabolized to serotonin (5-hydroxytryptamine)—a neurotransmitter that modulates neural activity and a wide range of neuropsychological processes like mood, perception, reward, anger, aggression, appetite, memory, sexuality, and attention [6]. In the pineal gland, serotonin serves as a precursor for the synthesis of melatonin, which is involved in the regulation of circadian rhythm, and the reproductive and immune systems [7]. Approximately, 3% of dietary TRP is utilized for serotonin synthesis throughout the body (1% is utilized in the brain) [8]. Alternatively, TRP might be converted to a trace amount of tryptamine—an important neuromodulator of serotonin [3]. About 95% of intake TRP is metabolized via the kynurenine pathway (KP), producing KYN and other derivatives [9]. KYN secretion by different cells plays an important role in immune privilege during infections, inflammations, pregnancy, and cancer. The contributions of the different metabolic pathways of TRP utilization may differ under physiological and pathological statuses.

Both excessive intake and deficiency of TRP can unbalance its homeostasis and affect human health [4]. The impact of TRP supplementation on human health has so far been studied mainly in terms of serotonin pathway activation. The benefits of TRP loading on human cognition, mood, and sleep as a result of serotonergic stimulation have been widely reported [10]. The KP is the main metabolic route of this amino acid. Moreover, food might contain KP metabolites itself, which has been confirmed by many studies [11,12,13,14,15,16,17,18,19]. Some of them are essential for the proper functioning of the body and have a beneficial effect, e.g., kynurenic acid (KYNA) or nicotinamideadenine dinucleotide. On the other hand, KP delivers some metabolites which could exert cytotoxic (e.g., KYN, 3-hydroxyanthranilic acid (3HAA), and 3-hydroxykynurenine (3HKyn)) and neurotoxic (e.g., quinolinic acid (QA)) impacts. Notably, elevated levels of some KP metabolites are associated with various diseases, e.g., increased KYN and KYNA are connected with inflammatory bowel disease [20] and ulcerative colitis [21], respectively. Providing compounds from KP with food can positively or negatively affect human health. Herein, we discuss the major functions of KP metabolites in the human body and summarize the data about their abundance in a variety of food commodities pointing to their potential external sources.

So far, many analytical protocols have been developed to study TRP and its downstream metabolites in biofluids. Among analytical techniques available, liquid chromatography (LC) connected with various detectors is a widely recognized modern and sophisticated analytical tool for monitoring TRP metabolism and reliable quantification of its intermediates in blood, plasma, urine, and cell supernatants [22,23,24,25,26]. In this review, we have also summarized current knowledge on LC-based methodological protocols serving for the quantification of KP metabolites in food samples.

## 2. Kynurenine Pathway—An Overview

**Figure 1:**
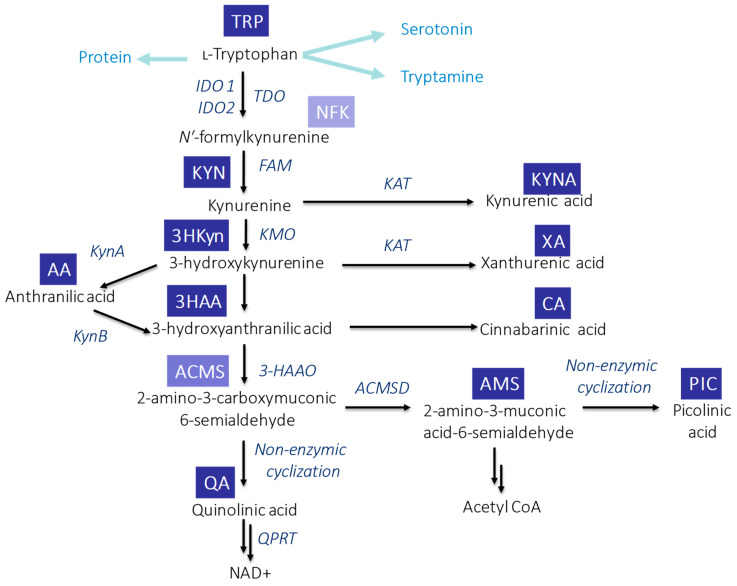
Schematic overview of TRP metabolism via KP in mammals. Abbreviations: ACMSD—2-amino-3-carboxymuconic acid semialdehyde decarboxylase; FAM—N′-formylkynurenineformamidase; 3-HAAO—3-hydroxyanthranilic acid 3,4-dioxygenase; IDO1—indoleamine-2,3-dioxygenase 1; IDO1—indoleamine-2,3-dioxygenase 2; KAT—kynurenine aminotransferase; KMO—kynurenine mono-oxygenase; KynA—kynureninase A; KynB—kynureninase B; QPR—quinolinate phosphoribosyl transferase; and TDO—tryptophan-2,3-dioxygenase.

In mammals, KP (Figure 1) is initiated through the activity of the enzymes: indoleamine-2,3-dioxygenases (IDO1 and IDO2) or tryptophan-2,3-dioxygenase (TDO). TDO acts mainly in the liver, while IDO has a wide tissue and cellular distribution. As a result of this reaction, N-formylkynurenine (NFK) is formed which is instantaneously converted by NFK formamidase to L-KYN—the first stable KP metabolite [27]. Degradation of L-KYN proceeds in three ways: (1) enzymes family of kynurenine aminotransferase (KAT) leads to the production of KYNA; (2) L-KYN can be hydrolyzed by kynureninase to anthranilic acid (AA); and (3) kynurenine mono-oxygenase (KMO) can lead to hydroxylation of L-KYN resulting in 3HKyn formation. The activity of some KP enzymes (IDO and KMO) is also upregulated by immune stimulation [28]. Further transformation of 3HKyn is accomplished by kynureninase and causes 3HAA formation [29]. The next product, 2-amino-3-carboxymuconic 6-semialdehyde (ACMS), is formed as a result of 3-hydroxyanthranilate-3,4-dioxygenase action. This unstable metabolite can be converted in two different ways: (1) preferably via QA, which, subsequently, undergoes the transformation and leads to the production of the essential redox cofactor nicotinamideadenine dinucleotide (NAD+) and (2) less efficiently 2-amino-3-muconic acid-6-semialdehyde (AMS), which, subsequently, generates picolinic acid (PIC) resulting from non-enzymatic cyclization or it is metabolized to acetyl CoA [27,30]. Alternatively, the transamination of 3HKyn by KAT leads to xanthurenic acid (XA) formation, whereas the dimerization of 3HAA produces cinnabarinic acid (CA) [28].

## 3. Circulation of KP Metabolites

In the human body, approximately <1% of ingested TRP is utilized for protein synthesis. TRP is transported by large neutral amino acid transporters mainly into the gut, where it is metabolized by microbiota [5]. The rest enters portal circulation and undergoes liver metabolism [31]. Notably, about 75–95% of circulating TRP is bound to albumin, but only free TRP can cross the blood–brain barrier (BBB). The unbounded TRP can be further metabolized along four degradation pathways [3,31].

Gut microbiota modulates intestinal TRP metabolism. Commensal microbes can transform TRP into tryptamine (by tryptophan decarboxylases), indole and its derivatives (by tryptophanase), and serotonin (by tryptophan synthetase). However, the initiation of TRP degradation via KP occurs by the activation of Toll-like receptors (TLRs) by microbial components. Notably, butyrate—produced by gut microbes—suppresses KYN production by downregulation of intestinal IDO expression via various mechanisms [32].

More than 60% of KYN is directly transported from peripheral circulation to the brain, where it is transformed into other neuroactive compounds [33]. 3HKyn and AA also have the ability to cross BBB, while KYNA, 3HAA, and QA cross it very poorly [33,34,35]. Furthermore, KYN can cross the placenta and fetal blood–brain barrier [36].

In peripheral tissues (liver and kidney), phagocytes (monocytes and macrophages), and microglia cells, KMO predominantly break down KYN to 3HKyn, which is further cleavaged to 3HAA and QA, leading to NAD+ formation [28]. Alternatively, KYN can be hydrolyzed to AA—a precursor for QA production. In astrocytes, however, KAT is catalyzed to KYN and converted to KYNA. Furthermore, glial cells and neurons produce PIC [28].

After oral ingestion, KYN is absorbed into the intestine [33]. Also, KYNA can be easily absorbed from the lumen of the digestive system into the bloodstream and transported to the liver and the kidneys [37,38]. In blood, its concentration achieves the highest level 15–30 min after ingestion, and back to the basal level after 2 h [38]. After ingestion, a successive increase in KYNA level in bile, pancreatic juice, and intestinal lumen is observed [39]. Under normal conditions, the majority of KP metabolites are excreted in the urine [39,40,41]. The presence of some KP metabolites in faeces and sweat was also demonstrated [39,41].

## 4. Biological Functions of KP Metabolites

The role of KP is connected with many diseases and conditions. The metabolism of TRP is related to neuropsychiatric and cardiovascular diseases, inflammation, and cancer, and KP metabolites have various biological functions (Table 1).

KYN shows immudulatory properties, e.g., it inhibits T-cell proliferation, reduces the activity of natural killer cells and dendritic cells, and promotes the differentiation of regulatory T-cells [46,47,48]. This metabolite shows antimicrobial activity and was identified as a specific agonist of the human aryl hydrocarbon receptor (AhR) [43]. Upregulation of KYN was noted in the case of such pathologies as infections, autoimmunological diseases, and cancer [49]. An increasing ratio of KYN/TRP is associated with cardiovascular diseases such as coronary heart disease [50,51] and it can be considered a biomarker for irritable bowel syndrome. Of note, it was demonstrated in rats, that this KP metabolite mediates vasorelaxation and lower blood pressure in a dose-dependent manner [52]. Furthermore, KYN production in the human body also varies when neurological and psychiatric disorders occur [53].

From a therapeutic standpoint, KYNA is one of the most interesting metabolites [28]. It is mainly produced in glial cells. It is also a competitive antagonist of glutamate receptors—NMDA receptors, kainate receptors, and AMPA receptors. The additional targets of KYNA are the α7 nicotinic acetylcholine receptor (α7nAChR), the former orphan G protein-coupled receptor (GPR35), and AhR [34]. KYNA shows neuroprotective, antioxidant-free radical scavenging properties, reduces oxidative stress, and decreases protein and lipid damage [28]. There are strong indications that this TRP metabolite exerts anti-inflammatory or proinflammatory effects, depending on whether inflammatory or homeostatic conditions are considered [54]. It was demonstrated on human natural killer T-cells that activation of GPR35 by KYNA (300 µM) significantly reduces the release of IL-4 [55]. This KP metabolite of ~500 µM dose suppresses the production of IL-23 and IL-17 by dendritic cells [56]. KYNA inhibits TNF-α release from human mononuclear leukocytes [57]. It was also demonstrated that cancer cells in the intestine produce KYNA more effectively in comparison with normal colon epithelial cells, without overexpression of KATs in these cell lines [58]. The antiproliferative action of KYNA (>10 µM) against in vitro cultured colon cancer cells (HT-29, LS-180, and Caco-2) has also been reported [58]. On the other hand, KYNA could influence intestinal inflammation and hypermotility [59]. An increased ratio of KYNA/TRP is associated with endoscopic inflammation and is predictive of disease outcomes in ulcerative colitis patients [21]. A higher level of KYNA was also noted in patients with schizophrenia relative to healthy controls (when KYNA content was analyzed in cerebrospinal fluid, central nervous system, and brain tissue samples) [60]. On the other hand, a decrease in KYNA occurs in patients with Parkinson’s and Huntington’s diseases, and multiple sclerosis [34].

3Hkyn and 3HAA are both good electron donors and their oxidation results in the formation of a highly reactive quinoneimine [42]. Even the micromolar concentration of these compounds contributes to the generation of the reactive oxygen species (ROS)—hydroxyl radicals and hydrogen peroxides—and induces oxidative stress [61]. This pro-oxidant behavior would explain some toxical actions of 3HAA and 3HKyn [47,62,63]. In particular, they are T-cell-suppressive and have an additive effect in the presence of KYN [47]. Furthermore, a large body of literature has demonstrated in vitro neurotoxicity of 3Hkyn by ROS overproduction, direct protein interaction, and mitochondrial dysfunction [42]. On the contrary, many reports reveal the antioxidant function of both 3HAA and 3HKyn [42,62]. For example, these two metabolites of 20 µM dose effectively protected B-phycoerythrin from peroxyl radical-mediated oxidative damage [64]. Increasing 3HKyn and 3HAA can be related to neurodegenerative disorders—Huntington’s, Parkinson’s, and Alzheimer’s diseases. Another KP metabolite—AA—could be considered a biological marker in schizophrenia [65] or gastric cancer patients [66]. Although AA is not efficient as a peroxyl radical scavenger, this KP metabolite acts as the chelating agent of Cu(II) ions and is a good protector against the hydroxyl radicals associated with oxidative stress through its secondary antioxidant activity [44].

QA is a well-known neurotoxin produced in the brain [45,67]. It can be a neuroprotective compound but only in low concentration. Increasing QA causes saturation of the catabolic system and this condition becomes toxic [68]. QA exerts neurotoxic effects by several mechanisms including activation of the N-methyl-d-aspartate (NMDA) receptor in pathophysiological concentrations [45]. Elevated levels of QA are noted to be in the case of patients with Alzheimer’s disease and inflammatory disorders. In Huntington’s disease, the increase in the brain QA is accompanied by an increase in cerebral 3Hkyn and a reduction in the brain KYNA [34]. Neurotoxicity of QA is associated with the pathogenesis of neurodegenerative processes connected with neuroinflammation [69]. Therefore, it is associated with such diseases as multiple sclerosis and Huntington’s disease. In vitro experiments demonstrated that QA does not induce the death of lymphocytes [47]. However, in TRP-free extracellular microenvironment, inhibition of T-cell proliferation could be observed [48].

KP by-products—XA and CA—are neuroactive compounds that modulate metabotropic glutamate (mGlu) receptors [28,70,71]. Studies performed on schizophrenia patients have shown a decrease in XA levels in serum compared to healthy controls [72]. This metabolite shows anticonvulsant and antioxidant properties [28]. The formation of XA is believed to be a major detoxification route for 3HKyn production [42]. Notably, CA is a ligand of AhR [70], and shows antibacterial properties [73].

3HAA, AA, and CA are also important players in the shikimic acid pathway [74], allowing the biosynthesis of aromatic amino acids in fungi, bacteria, and plants.

PIC is utilized for the production of local anesthetics or supplements [75], shows antiviral, antifungal, antitumoral properties, and regulates cell growth. In the human body, this compound plays the role of a chelating agent of such elements as zinc, copper, iron, molybdenum, and manganese [76]. It inhibits the proliferation of peripheral blood leukocytes [48].

## 5. Tryptophan Supplementation and Generation of KP Metabolites

Mammals cannot synthesize TRP which has to be supplied by ingested proteins. In the Caucasian diet, milk and sour-milk products, meat, beans, beets, and nuts are good sources of this amino acid. In the Mediterranean diet, TRP might be supplied with whole grains, nuts, and fish. The estimated average requirement (EAR) of TRP by the WHO is set at 4 mg/kg per day for adults (about 280 mg/day for a 70-kg adult) [77]. However, the estimated mean individual intake of TRP by adults might be higher than EAR (e.g., 826 ± 3 mg/day among US adults) [78]. Consuming higher doses of TRP has some beneficial effects on human health, e.g., decrease in a depressive mood, improvement of perceptual-motor and vigilance performance in low chronic stress-vulnerable subjects [79], and improvement in the quality of sleep duration [78,80]. All of these observations can be explained by increased plasma TRP for uptake into the brain and higher serotonin production. In the brain, however, TRP can be depleted via KP, thus, astrocytes, microglia, brain-infiltrating macrophages, and dendritic cells express IDO. Altered KYN metabolism and generation of downstream metabolites (neurotoxic 3HKyn, 3HAA, QA, and neuroprotective KYNA) within the central nervous system have been implicated in the pathophysiology of Huntington’s disease, Alzheimer’s disease, and acquired immunodeficiency syndrome (AIDS)-related dementia [81]. On the other hand, low TRP intake can cause disorders like pellagra resulting from nicotinic acid deficiency [82]. Furthermore, some long-term studies have demonstrated no changes in mood in healthy controls [83] or in individuals with depressed mood or risk of suicide [84] after increased TRP intake during dieting. It was also observed that in people with untreated depression, TRP depletion tends to have no mood effect, unlike patients who have responded to antidepressants [85].

Generally, oral TRP supplementation is accompanied by an increase in urinary excretion of KP metabolites [86]. A similar effect is observed after the administration of hydrocortisone, which induces TDO toward TRP depletion [86].

The role of dietary TRP on the immune system has also been investigated, while TRP catabolites via KP show immunomodulatory functions. This metabolic route of TRP is usually systematically upregulated under immune activation and inflammation, which is observed among others in obesity. A decrease in serum TRP and KYN in obese people after a calorie-restriction weight loss diet has been reported. However, these changes can be referred to lower TRP intake during a calorie-restriction diet, because no changes in the immune activation status of individuals have been observed [87]. Also, no significant changes in urinary excretion of TRP and its metabolites (including KYN, 3HKyn, 3HAA, KYNA, XA, QA, and AA) in Japanese healthy women after 22 days of TRP administration have been reported [88]. Another study conducted on Japanese young women has shown no changes in food intake, body weight, general biomarkers, and amino acid composition in blood and urine after oral administration of different doses of TRP (from 1 to 5 g/day for 21 days) [89]. Blood levels of KP metabolites (KYN, KYNA, AA, 3HKyn, XA, and 3HAA) were very low, trace, or undetectable, rendering it impossible to study changes in their concentrations after TRP supplementation. On the other hand, the urinary excretion of KP metabolites (including KYN, AA, KYNA, 3HKyn, 3HAA, QA, and XA) increased in proportion to the ingested amounts of TRP. Urinary excretion of 3HKyn was abnormal after TRP administration, thus, 3HKyn has been suggested as a surrogate indicator for preventing and predicting L-TRP toxicity. In another study, the urinary excretion of major TRP metabolites in Japanese healthy women after 1, 7, 14, and 21 days of TRP administration has been investigated [90]. In light of this study, the urinary excretion of some KP metabolites (KYNA, 3HKyn, XA, and 3HAA) was increased on day 7, and then remained constant on days 14 and 21 of TRP supplementation. KYN and AA concentration in urine was low. The researchers concluded that TRP metabolites do not accumulate in the body.

## 6. Fate and Health Effects of KP Metabolites after Oral Administration and Food Intake

The intravenous infusion of KYN was found to be safe for humans and well tolerated up to a dose of 5 mg/kg [91]. Another study, conducted on a group of normal post-menopausal women, has demonstrated that KYN sulphate intake (700 µmol) resulted in a substantially increased urinary excretion of KYN, 3HKyn, and 3HAA accompanied by a slight increase in KYNA and XA [86]. On the contrary, TRP (9800 µmol) load led to a significant increase of KYNA and XA in urine. These observations are consistent with the results obtained in a similar study on women using an estrogen-containing oral contraceptive [92]. Furthermore, studies on rats indicated that KYN does not accumulate in blood plasma and liver tissue up to 24 h after intake [93]. The adverse effect was observed for KYNA, which does not undergo extensive metabolism [94]. KYNA administration (25 or 250 mg/kg) resulted in a steady-state increase of this KP metabolite content in rats’ serum, the liver, and the kidneys lasting for at least 2 h after drug intake [38]. Intake of high doses of KYNA (250 mg/kg) was followed by the increase of its level to micomolar level, which is sufficient to interact with GPR35, NMDA, and α7nAChR receptors [38]. In another study on rats, the maximum increase in KYNA level in the liver was noted at 1 h of its administration at 10 mg/kg dose (the sustained high KYNA remained over the subsequent 3 h) [94]. Furthermore, 3Hkyn, 3HAA, and AA also increased in the rat liver by most of the KYNA doses within the range of 1–10 mg/kg at 1 h of administration [94]. Notably, the changes in levels of KP metabolites in the liver did not reflect those observed for corresponding serum samples [94]. The study on male and female healthy volunteers eating chestnut honey containing KYNA in an amount of 600 mg/kg has shown an increase of KYNA in serum 30 min after administration indicating its absorption from the digestive tract [14]. On the other hand, performed studies have shown that long-term consumption of water containing KYNA by rodents did not cause a toxic response [95]. The wide range of health-promoting effects of KYNA in animals, respecting different routes of administration, was summarized in detail in the review [39]. They include cholesterol-lowering, hepatoprotective, wound healing, antiatherosclerotic, anti-inflammatory, antimigraine, antioxidant, antiobesity, antiosteoporotic, antiulcerative, antiviral, and probiotic stimulating effects.

Differences in the fate of KYN and KYNA after oral intake have also been demonstrated in animal experiments. For instance, oral administration of KYN or KYNA in pregnant mice raised the level of both metabolites in maternal plasma and placenta [36]. Unlike KYNA supplementation, the maternal administration of KYN resulted in an increase of KYNA and 3HKyn in the placenta, fetal plasma, and brain. During breastfeeding, KYN and KYNA in breast milk are modulated and these two KP metabolites can affect differently the offspring postnatal maturation [13,93]. An experiment performed on rats exposed to KYNA-enriched diet during the time of breastfeeding, indicated KYNA increase in maternal milk [13]. KYNA can act as an antiobesogen since a reduction of body weight of both rats postnatally exposed to KYNA supplementation and rat offspring fed with breast milk enriched with KYNA was observed [13]. On the other hand, no weight gain in rats administered KYN compared with those fed a normal diet was noted [93]. KYN supplementation during the suckling period, however, was connected with behavior changes in adult rats—reduced susceptibility of female rats to the action of dizocilpine and enhanced susceptibility of male rats to the action of amphetamine [93]. Furthermore, impaired cognitive flexibility in the contextual fear conditions in male rats was observed. Simultaneously, KYN supplementation during the suckling period did not influence the spontaneous locomotor activity, recognition memory, and anxiety-like and depressive-like behavior in adult rats [93]. Similar observations were made during the study on KYN-supplemented mice [96]. Importantly, both KYN and KYNA are agonists of AhR, whose presence in the digestive tract of young breast-fed animals was confirmed [93].

It was suggested that NFK, KYN, and KYNA could, potentially, be involved in red meat-associated diseases such as cardiovascular diseases [97]. This assumption was supported by untargeted metabolomic analysis of chicken and beef digestion samples obtained by incubation of meat with the fecal inocula of healthy volunteers. It was also demonstrated in humans that regular consumption of tea over 6 months increases blood KYN without significant changes in TRP. The observed increased KYN production in individuals with major illnesses has been linked to blood pressure lowering, which could be beneficial for cardiovascular health [98].

In rats, an effect of oral supplementation with 3HKyn and 3HAA of a 10 mg/kg dose on TRP and KP metabolites levels in the liver was also investigated [94]. The maximum increase in liver 3HKyn and 3HAA was noted after 1 h. However, 3Hkyn remained high over the next 2 h, but 3HAA further increased at 4 h after administration. Furthermore, the acute administration of 3HAA was accompanied by an increase in hepatic KYN and KYNA, a steady decrease in AA, and an enhancement of TDO activity. On the other hand, administration of 3HKyn did not influence significantly liver KYN and TRP, and TDO activity as well [94].

## 7. Occurrence in Food Products

Food is a source of TRP and its metabolites deriving, among others, from KP. Due to the various properties of these compounds, their occurrences have been checked and they have been willingly quantified in food products. It is an important aspect to cognize sources of exogenous TRP metabolites and their possible influence on human health.

Among different KP metabolites, KYNA is mainly supplied to the human body with food [37], and its content was predominantly studied in various feeds and foods. It is believed to have positive properties when it comes to the gastrointestinal tract [19]. However, the KYNA-reach diet could be unsuitable for patients with ulcerative colitis, since the increased KYNA/TRP ratio is associated with endoscopic inflammation and predictive of disease outcomes [21]. This case reveals that the functional consequences of dietary KYNA in this setting merit further study. The role of dietary KP metabolites in patients with gastrointestinal cancer or cardiovascular diseases also needs further investigation.

This section summarizes the results of the examinations of the contents of TRP and KP metabolites in different foods and beverages. In the literature data, they have been determined individually or as a panel of several substances.

### 7.1. Herbs and Species

The presence of KYNA was broadly studied in various herbs and spices, whereas other KP metabolites are frequently not evaluated (Table 2). Despite the leaves of basil being characterized by the highest amounts of KYNA (~14 mg/kg), its contents in the roots of dandelion and Harpagophytum or fruits of black pepper are much lower (≤0.1 mg/kg). Thyme leaves and branches and leaves of hemp (*Cannabis sativa* L.) also contain relatively high KYNA levels (~8–10 mg/kg). Notably, KYN levels in different parts of hemp (leaves, stem, and roots) are much higher than KYNA levels (Table 1). Analysis of herbs and species in terms of determining other KP metabolites warrants investigations.

### 7.2. Fruits and Vegetables

Various popular vegetables and fruits were intensively studied in terms of KYNA (Table 3). Broccoli was found to be rich in this compound (0.4 mg/kg). However, considering daily intake, potato tubers might be an important source of this metabolite in everyday diet, since they contain from 0.24 to 3.24 mg/kg dry weight depending on the variety. Potato-related foods (French fries, crisps, and flour) contain lower amounts of KYNA (Table 3). In potato tubers, KYNA decreases with the time of storage, and does not depend on starch content, maturity, or storage ability [17]. Notably, boiling does not affect KYNA in potatoes, unlike in carrots, cauliflower, and broccoli (drop by 37, 81, and 88%, respectively) [38]. Other KP metabolites were sparsely studied in fruits and vegetables (Table 3). Roasted soybean could be a better dietary source of KYN and AA than of KYNA.

### 7.3. Mushrooms

Edible mushrooms are a source of both hallucinogenic and non-hallucinogenic indole derivatives, including TRP metabolites such as serotonin, melatonin, and KYN. Muszyńska et al. [102] prepared methanolic extracts of the fruiting body and mycelium of *Cantharellusci barius* (the chanterelle), while mycelium was cultured in vitro. Obtained extracts were analyzed in terms of TRP and its metabolites. Of the substances determined, only KYN was derived from KP. The content of KYN was high in both the fruiting bodies and mycelium, while in mycelium it was significantly higher (about 10 times) and equaled about 36.2 mg/kg and 353.4 mg/kg, respectively [102]. Attempts have also been made to determine TRP metabolites in other edible mushrooms but detectable amounts of KYN sulphate were noted only in *Suillus luteus* (~26.3 mg/kg), *Boleus badius* (~19.6 mg/kg), *Lactarius deliciosus* (~392 mg/kg), and KYNA in *Boleus badius* (~15.7 mg/kg) [103,104]. However, the release of KYN from the fruit body of selected edible mushrooms (*Agaricus bisporus*, *Boletus badius*, and *Cantharellusci barius*) to artificial stomach juice was not detected [105]. Furthermore, the thermal processing of some mushrooms did not favor the generation of KYN and KYNA [104]. Notably, inedible *Pycnoporus cinnabarinus* is recognized from the orange–red color of the fruit body caused by CA produced from 3HAA [73].

### 7.4. Honey and Honeybee Products

Literature data confirmed that honey is a rich source of KP metabolites. Especially, the quantification of KYNA was performed by researchers due to its beneficial properties for human health [14,38]. The content of TRP, KYN, and XA was also tracked [15], but other KP metabolites were omitted from the target list (Table 4). Diverse types of honey (e.g., rosemary, thyme, and sunflower) and some honeybee products (bee pollen and propolis) were analyzed. Chestnut honey is distinguished by a high KYNA, while sunflower, thyme, and eucalyptus honeys contain elevated KYN compared to other honey types.

### 7.5. Fermented Products

Due to the beneficial health effects of fermented products, which are associated with the presence of microorganisms and their metabolites, their popularity as a part of the human diet has been increasing rapidly. TRP metabolites from KP occur in fermented products due to the metabolism of bacteria and yeasts. *Saccharomyces cerevisiae*, *Saccharomyces uvarum*, and *Saccharomyces pastorianus* (yeast utilized in the production of beer and bakery products) [106,107,108,109], and prokaryotes such as *Cyanidium caldarium*, *Karlingiarosea*, *Streptomyces antibioticus*, and *Xanthomonas pruni* utilize TRP via KP [110]. This assumption was confirmed by others [18].

#### 7.5.1. Milk and Milk-Based Fermented Products

TRP and its KP metabolites occur in milk and milk-fermented products such as yogurt, kefir, cheese, and probiotic drinks (Table 5). TRP concentration in food increased with fermentation. It was also noted that KYN depends on the milk temperature [111]. By increasing temperature, the content of KYN in raw sera decreases (e.g., about 0.2 mg/L loss of KYN after heating to 143.3 °C was observed). Probiotic products contain high TRP content probably due to the proteolytic action of added cultures [16]. Based on already gathered data, it could be assumed that amounts of KYN in milk-based fermented products are higher than KYNA (Table 5). Furthermore, human milk might contain higher KYNA amounts than 1.5% cow’s milk (25.7 and 17.4 µg/kg, respectively) [38]. It is worth mentioning that KYN and KYNA in human milk gradually increase over the first month after post-partum and in the first weeks of lactation [13,40] (see Section 7.6). Data about the concentrations of other KP metabolites in this type of food are missing.

#### 7.5.2. Traditional Fermented Food

Kinema is a traditional fermented soybean food with a slight ammoniacal flavor of the Eastern Himalayan regions of North East India, east Nepal, and South Bhutan. This sticky, slightly alkaline product is produced by the natural fermentation of “yellow cultivar” soybean seeds during 1–3 days. The analysis of kinema samples collected from India, Nepal, and Bhutan indicated the presence of 3HKyn [112]. However, further targeted investigations are needed to assess whether kinema or other traditional fermented foods might be a dietary source of other KP metabolites.

**Table 5:** Occurrence of TRP and its KP metabolites in milk and fermented products. 3HAA, AA, 3HKyn, XA, CA, PIC, and QA were not included in a target list of the analytical methods utilized for quantitative analysis.

Product	N	Concentration Range (mg/kg) or (mg/L)			Ref.
		TRP	KYN	KYNA	
Bread	3	31.70–72.50	<LOD–0.14	<LOD	[18]
	–	n.a.	n.a.	0.01	[38]
Cheese (white)	6	3.80–37.60	0.03–0.32	0.03–0.08	[18]
Cheese (hard)	–	n.a.	n.a.	0.008	[38]
Milk	11	~0.30	~0.08	n.a.	[16]
Milk (1.5%)	–	n.a.	n.a.	0.02	[38]
Milk (human)	–	n.a.	n.a.	0.03	
	24	~200.0–300.0 a	~0.02–0.07 a	~0.01–0.04 a	[113]
	–	n.a.	n.a.	0.004–0.06 b	[13]
	82-88	0.22–10.90 c	0.01–0.22 c	n.a.	[93]
Kefir	3	2.90-5.40	0.37–0.76	0.11–0.24	[18]
	–	n.a.	n.a.	0.01	[38]
Probiotic drinks	31	0.22-5.16	~0.06	n.a.	[16]
Yoghurt	5	3.20-13.40	0.29–0.75	0.07–0.29	[18]
	9	~0.70	~0.07	n.a.	[16]
	–	n.a.	n.a.	0.02	[38]

a—samples collected on day 7 and day 14 of post-partum; b—samples collected during the first 6 months after post-partum; c—samples collected during the first 4 weeks of lactation; n.a.—data are not available, the analyte was not included in the target list of the applied analytical methods; <LOD—not detected; and N—number of analyzed samples. AA—anthranilic acid; CA—cinnabarinic acid; 3HAA—3-hydroxyanthranilic acid; 3HKyn—3-hydroxykynurenine; KYN—kynurenine; KYNA—kynurenic acid; PIC—picolinic acid; QA—quinolinic acid; TRP—tryptophan; and XA—xanthurenic acid.

#### 7.5.3. Beer

Kinetic evaluation of the formation of KP metabolites during wort fermentation indicated that ale yeast (*Saccharomyces cerevisiae* NCYC 88) utilizes more TRP than lager yeast (*Saccharomyces pastorianus* NCYC 203) [109]. Both yeast species can produce KYN and KYNA from TRP [109]. In *Saccharomyces cerevisiae*, the following enzymes, Bna2 (tryptophan 2,3-dioxygenase), Bna7p (NFK formamidase), Bna3p (KAT), and Bna5 (kynureninase), which allow for the conversion of TRP to NFK, NFK to KYN, KYN to KYNA, and KYN to AA, respectively, have been identified [109,114]. Furthermore, other KATs—Aro8 and Aro9—were reported to be responsible for the formation of KYNA from KYN [109,115]. It was also observed that the initial concentration of TRP strongly influences the rate of KYNA production during fermentation. KYNA concentration increases up to day 12 of fermentation but the rate of its production depends on the yeast strain utilized [109]. According to literature data, the estimated daily intake of KYNA with beer is 2.6 µg/day [39].

#### 7.5.4. Wine

Although beer was found to be the richest source of TRP, red wine contains the highest amount of KYNA [18]. The concentration of TRP metabolites (KYN, PIC, 3HKyn, and QA) changes during wine fermentation [116]. It was confirmed by analysis of samples of white wine (Sensy, Flavia, Melody, and large-scale white wine) at different times of fermentation using non-*Saccharomyces* and *Oenococcus oeni* yeasts [116]. Only 3HKyn was not detected in analyzed wines (Table 6). KYN was detected only in two types of wine—Sensy and Flavia wine—but only after 4 and 5 days of fermentation, respectively. In all types of wine, KYNA was detected from the beginning of fermentation. In large-scale white wine, the level of KYNA was similar throughout the fermentation time, while, in the case of other wines, the concentration increased over time. The presence of PIC and QA in wine samples was also confirmed [116]. The content of PIC varied on different days of fermentation ranging from 22.4 to 70.2 µg/L. The highest level of PIC was noted in Melody wine. QA was not detected in large-scale wine, while the other wines contain this substance at the level from 24.7 to 43.9 µg/L after 5 days of fermentation. Forino et al. determined another metabolite of TRP—XA in red wine (Aglianico wine) [117].

#### 7.5.5. Other Alcoholic Beverages

Other types of alcoholic beverages like cider (made from the fermented juice of apples), vodka (obtained usually from fermented grains or potatoes), cognac (prepared using fermented grapes), whisky (made of a mash of fermented grains), liqueur (made of grains, fruits, or vegetables), and mead (obtained by fermenting honey) were screened for KP metabolites (Table 6). Unlike mead, only small amounts of KYNA were detected in distilled beverages [11].

### 7.6. Breast Milk and Baby Formulas

Nowadays, much interest is paid to the safety of baby formulas. Breast milk, which is the only source of TRP in breast-fed infants, also contains KYN and KYNA [13,113]. Milart et al. analyzed the content of KYNA in breast milk (from 25 healthy breast-feeding women in the period from the 3rd day to the 6th month) and compared its concentration with that found in artificial nutritional formulas dedicated to babies from birth to over six months. In both breast milk and artificial baby formulas, the presence of KYNA was confirmed, but its content significantly differed. The interesting finding was that KYNA in human milk from women in the 3rd day to the 6th month breastfeeding period increased from 3.9 ± 0.6 µg/L to 41.5 ± 5.5 µg/L. On the other hand, artificial nutritional formulas dedicated to babies from 0 to the 3rd month contain significantly less KYNA (5.0 ± 0.7 µg/L). Similarly for older babies: baby formulas dedicated to babies from the 4th to the 5th month contained KYNA at the level of 4.8 ± 1.0 µg/L, while human milk is richer in KYNA and contains from 43.1 ± 4.8 µg/L (the 4th month of the breastfeeding period) to 47.5 ± 8.9 µg/L (the 5th month of the breastfeeding period) of this TRP metabolite. In human milk in the 6th month of the breastfeeding period, KYNA reaches a concentration of 56.6 ± 8.6 µg/L and in artificial formulas for the over-6-months-of-life period, only 7.3 ± 0.9 µg/L. In the performed research, individual artificial nutritional formula brands of five producers were studied in terms of KYNA content [13].

KYN content was evaluated in human breast milk during four weeks of lactation and infant formulas were utilized for feeding during the postnatal time [93]. In breast milk, KYN has increased in time (from 4 to 28 days of breastfeeding) from 31 to 81 µg/L (based on median values). In baby formulas (commercially available powdered (*n* = 9) and liquid (*n* = 2) samples), KYN was significantly higher and ranged from 20–28 mg/L. In another work performed on infant formulas, KYN was found in the range of 0.02–15.0 mg/kg [118].

Human milk contains significantly more KYNA than baby meals, which contain this metabolite at a concentration within the range of 0.96–14.80 µg/kg [38]. To the best of our knowledge, the determinations of other KP metabolites in baby foods have not been conducted so far.

### 7.7. Tea and Coffee

It turned out that TRP metabolites can also be supplied with beverages (Table 7). An example of KYNA sources is tea and coffee [12]. The analysis of 16 types of tea and rooibos from different countries of the world revealed that each tea extract (which was prepared according to the producers’ recommendations) contained KYNA. Its amount in 100 mL of tea extract varies from 0.5 µg to 8.7 µg (in Che NhaiDacBiet Jasmine green tea from Vietnam and Ginkaku Sencha green tea from Japan, respectively). Similarly, the presence of KYNA in coffee was found in all tested coffee samples. Its concentration was in the range of 0.02–0.63 µg/100 mL. The whole beans of Dallmayr Standard 100% Arabica characterize the lowest content of KYNA, while the highest was in instant coffee Tchibo Exclusive 100% Tchibo Arabica [12].

Extensive research on the content of KP metabolites (KYN, KYNA, 3HKyn, PIC, and QA) in Oolong tea (with different oxidizing times), green tea, and black tea has been conducted [119]. Additionally, the changes in contents of TRP metabolites in tea were tested after a few different oxidizing times (10, 30, and 60 min). In all types of tea, only the content of KYNA, KYN, and QA was detectable. The highest concentration was noted for KYNA, from 1 to 3 mg/kg. Depending on the oxidizing time, the content of KYN varied from 0.05 to 0.6 mg/kg. QA content in all teas was the lowest and ranged from 0.1–0.3 mg/kg. Generally, the content of all determined TRP metabolites was higher in green tea than in black and varied depending on the shooting period [119].

### 7.8. Meat

Available data are limited to KYN and KYNA. Screening analysis of raw poultry meat was conducted to evaluate KYN and KYNA [120]. For the examination, two types of meat were chosen—chicken and turkey—and meat samples (89 samples in total) derived from different parts of the body (breast, drumstick, thigh, mid joint wing, back, and neck). A detectable amount of KYN was observed in 85% of the total samples and its range was 0.013–0.27 mg/kg. The study has revealed that turkey meat is a richer dietary source of KYN than chicken. KYNA was determined at the lowest concentration level (0.004 and 0.14 mg/kg) and only in 3% of samples was its concentration quantifiable [120]. Meat from beef (0.09 mg/kg), pork (0.10 mg/kg), and fish (roach, 0.04 mg/kg) contains comparable amounts of KYNA, while pig (liver, 0.26 mg/kg) is richer in this metabolite [38]. The low contents of KYNA in meat could be explained by its rapid extraction from the animal’s body [39].

## 8. Chromatographic Determination of KP Metabolites in Food Commodities

Because of the involvement of TRP metabolites in many diseases, they have been generally determined in biological samples such as cells, tissues, plasma, serum, and urine. Undoubtedly, LC coupled with various detectors has become the core analysis technique in studies focused on the determination of KP metabolites in complex biological media [24]. Indeed, other analytical techniques can be utilized for this purpose [46]. However, in the case of quantification of TRP derivatives in food samples, there is a limited number of papers dealing with the topic. Nevertheless, LC has also found an application in studies investigating KP metabolites in food products.

According to the literature data, KYNA is one of the most studied KP metabolites in foods. This metabolite forms a fluorescent adduct with zinc ions and may be determined at nanomolar concentrations in biological samples using high-performance liquid chromatography connected with a fluorescence detector (HPLC-FD) [24]. Derivatization may be achieved on-column using a mobile phase of pH 6.2 containing 250 mmol/L of zinc acetate. KYNA signal is measured by setting the excitation and emission wavelength at 344 and 398 nm, respectively. This strategy has been adopted in food research for KYNA determination in alcoholic beverages [11], culinary herbs and spices [19], potato tubers, crips, French fries [17], flour, honeybee products [14], and milk [13]. KYNA can be also determined using high-performance liquid chromatography connected with a diode array detector (HPLC-DAD), which was demonstrated by Soto and co-workers [15]. In that work, TRP was detected using the fluorescence detector (276 and 354 nm excitation and emission wavelengths, respectively), whereas KYN at 240 nm KYNA and XA at 226 nm by DAD. The method was applied for the determination of the analyte in honey samples. The obtained limits of detection (LODs) were from 5 to 9 µg/kg for TRP, from 18 to 36 µg/kg for KYN, from 5 to 16 µg/kg for XA, and from 4 to 18 µg/kg for KYNA depending on the botanical origins of the sample. Quantification of TRP and KYN in milk-based products [16] utilizing HPLC connected with different ultraviolet detectors has also been reported.

Nowadays, the use of a mass analyzer in combination with LC addresses current hot issues in multi-target analysis of organic contaminants and residues in food commodities. Soto and coworkers have reported that LC connected with atmospheric pressure chemical ionization mass spectrometry (LC-APCI-MS/MS) provides lower LODs of TRP, KYN, KYNA, and XA in the honey matrix compared to HPLC-DAD/FD [15]. These analytes can also be simultaneously determined in food using an electrospray ionization (ESI) source connected with a tandem mass analyzer (LC-ESI-MS/MS) by monitoring protonated ions. LC-ESI-MS/MS has also found application in the determination of TRP and its derivatives in various fermented foods like beer, red wine, bread, yogurt, white cheese, kefir, cocoa powder [18], or tea [119]. LC coupled with other mass analyzers (quadrupole time-of-flight (Q-TOF) or single quadrupole (SQ) mass spectrometers) operated in positive polarity has been successfully applied for the determination of TRP and KP metabolites in infant formulas [118] or poultry meat samples [120].

## 9. Sample Preparation for Chromatographic Determination of KP Metabolites in Food—Practical Aspects

Even with the widespread use of LC-MS/MS in the quantitative analysis of foods, it suffers from the so-called matrix effect. Co-eluting compounds present in the matrix can interfere with the target analyte in the ion source causing a loss in response (ion suppression) or an increase in response (ion enhancement). The matrix effect can be caused by sample components released during the sample preparation step or reagents added to the mobile phase to improve chromatographic resolution. Alongside available ionization methods, ESI has found numerous applications in the determination of TRP metabolites in a variety of samples. Although ESI ensures excellent results in the analysis of substances of low molecular mass, the method suffers strongly from matrix effect. Strong ion suppression was noted, e.g., during KYN and KYNA determination in poultry meat [120] or TRP and KYN in infant formulas [118]. Atmospheric pressure chemical ionization (APCI) shows less matrix effect than ESI. Nevertheless, strong matrix effects during TRP, KYN, KYNA, and XA determination in honey samples by LC-APCI-MS/MS have been reported [15]. Matrix effects can hamper both the identification and quantification of an analyte, thus, efforts should be devoted to evaluating, minimizing, and/or compensating for them. Using internal standards (structural analogs and stable isotopically labeled compounds) leads to compensation of matrix effects, but does not avoid the loss in sensitivity. Standard addition or matrix-matched calibration curve methods are common approaches for matrix effect compensation. However, these methods have some limitations. The standard addition approach requires separate calibration for each sample. Calibration using the sample matrix-matched curve should be conducted for each matrix and is limited to a suitable blank sample. Furthermore, both approaches are time-consuming and labor-intensive. Nevertheless, none of the methods listed above can work without proper sample preparation, which plays a vital role in reducing matrix effects and obtaining reliable results.

Because each matrix has a unique composition, sample preparation protocols can differ depending on sample type, research goals, or applied analytical method. Widely utilized sample preparation methods like liquid–liquid extraction (LLE), protein precipitation, filtration, centrifugation, dilution, solid phase extraction (SPE), QuEChERS (quick, easy, cheap, effective, rugged, and safe) can be utilized for the determination of TRP and KP metabolites in a variety of foods. If sample preconcentration is needed, we should be aware that the concentration of both analytes and interferences increases. This can be accomplished by enhancing the matrix effects, too. On the other hand, sample dilution to diminish matrix effects could fail in the case of the determination of KP metabolites, because their amounts in food are generally low. Protein precipitation results in supernatant reach in many residual matrix components, which can lead to a significant matrix effect [121]. It was demonstrated that a simple sample pretreatment by protein precipitation provides more nonvolatile solute in the final extract compared to SPE and LLE. These nonvolatile sample components have been suggested to cause the loss of ESI response associated with ionization suppression [122]. Furthermore, a type of organic solvent utilized for protein precipitation has a big impact on the cleanliness of the final extract. For instance, it was demonstrated that protein precipitation with methanol results in an extract containing significantly more phospholipids compared to acetonitrile [121]. Endogenous phospholipids cause matrix effects in an MS source. Further extract purification using SPE is a good method of removing unwanted impurities that cause ion suppression, such as phospholipids, proteins, lipids, and salts. For lipids removal, sample pretreatment using EMR-Lipid—a product designed for selective capture of the long hydrocarbon chain of lipids—can be considered [118].

Traditional extraction techniques, like LLE or Soxhlet extraction, provide a relatively large amount of sample, but they are time-consuming, labor-intensive, and require high solvent consumption. QuEChERS is one of the modern analytical techniques, which was developed for pesticide residue analysis in food. The method consists of sample extraction by shaking with solvent (acetonitrile), salt-out portioning of water with a mixture of salts, and, eventually, cleanup using dispersive solid phase extraction (dSPE). The flexibility of QuEChERS has rendered this technique useful in many fields of food research and can be involved (after modifications) in the determination of KP metabolites in the food matrix [118]. Regarding the differences in the behavior of KP metabolites during the extraction step and a broad concentration range of TRP metabolites within one matrix, the sample preparation step should be carefully optimized considering the target analyte and research aim.

## 10. Conclusions

Tryptophan is an essential amino acid that has to be supplied by food. It is incorporated into body protein or is depleted to intermediary metabolites associated with numerous physiological functions. Although some tryptophan catabolites show health benefits, others are known to show cytotoxic or neurotoxic actions. Data indicate that kynurenine pathway metabolites contribute to autoimmune diseases, mood disorders, obesity, and cancer. So far, only a few attempts have been performed to investigate the safety of oral intake of kynurenine and kynurenic acid. The already gathered data suggest that kynurenine and kynurenic acid supplied with food would not affect negatively human health, but further investigations need to be conducted before any conclusions are derived. Furthermore, the fate of different kynurenine pathway metabolites after oral administration or food intake on human health is still poorly known and warrants further investigations. Another issue that should be explored is whether metabolites absorbed from the gut would be physiologically relevant. The interactions between the endogenous and exogenous kynurenine pathway metabolites and their mutual contribution in different physiological or pathological states (e.g., on inflammatory and stress responses) also merit consideration. The role of dietary kynurenine pathway metabolites in the pathogenesis of various bowel syndromes and gastrointestinal cancer needs also clarification. A closer look at this topic can lead to interesting conclusions or set new directions for further research.

## Figures and Tables

**Table 1:** Comparison of positive and negative health effects of the KP metabolites.

Biological Effect	KP Metabolite	Ref.
Anticonvulsant properties	KYNA, PIC	[28]
Anti-inflammatory properties	3HAA, 3HKyn	[28,42]
Antimicrobial activity	KYN, KYNA, CA, PIC	[28,43]
Antioxidant properties	KYNA, XA, AA, 3HKyn	[28,42,44]
Antiviral properties	PIC	[28]
Immunomodulation	KYN, CA	[28,43]
Lipid peroxidation	QA	[45]
Neurotoxicity	3HKyn, QA	[42,45]
Neuroprotective properties	KYNA	[28]
Oxidative stress regulation	KYN, 3HKyn, 3HAA	[28]
Proconvulsant properties	QA	[28]
Pro-oxidant properties	3HKyn, QA	[28,42]
Reduction of lipid peroxidation	KYNA	[28]
Transcription factor	KYN	[28]
Vasodilator in endothelial cells	KYN	[28]

AA—anthranilic acid; CA—cinnabarinic acid; 3HAA—3-hydroxyanthranilic acid; 3HKyn—3-hydroxykynurenine; KYN—kynurenine; KYNA—kynurenic acid; PIC—picolinic acid; QA—quinolinic acid; and XA—xanthurenic acid.

**Table 2:** The concentrations of TRP and some KP metabolites in herbs, species, and herb-based diet supplements. 3HKyn, XA, CA, PIC and QA were not included in a target list of the analytical methods utilized for quantitative analysis.

Herbs/ Species	Form	Concentration Range (mg/kg)					Ref.
		TRP	KYN	3HAA	AA	KYNA	
Basil	leaves	n.a.	n.a.	n.a.	n.a.	12.75–15.41	[19]
Bay leaf	leaves	n.a.	n.a.	n.a.	n.a.	0.90–0.92	
Bean	pericarp and loose herbs	n.a.	n.a.	n.a.	n.a.	0.50–0.64	[99]
Birch	loose herbs	n.a.	n.a.	n.a.	n.a.	2.28–3.08	
Black pepper	fruits	n.a.	n.a.	n.a.	n.a.	0.09–0.11	[19]
Cloves	flower buds	n.a.	n.a.	n.a.	n.a.	1.22–1.36	
Cocoa	powder	12.9–37.5	<LOD	n.a.	n.a.	4.0–4.5	[18]
Cumin	seeds	n.a.	n.a.	n.a.	n.a.	0.61–0.67	[19]
Curry	powder and mixture of herbs	n.a.	n.a.	n.a.	n.a.	3.13–3.65	
Dandelion	root and loose herbs	n.a.	n.a.	n.a.	n.a.	0.04–0.06	[99]
Elder	flower and loose herbs	n.a.	n.a.	n.a.	n.a.	1.54–1.92	
Fennel	fruits	n.a.	n.a.	n.a.	n.a.	0.70–0.90	[19]
Glechoma	herbs	n.a.	n.a.	n.a.	n.a.	0.99–1.05	
Harpagophytum **	root and capsules	n.a.	n.a.	n.a.	n.a.	0.02–0.04	[99]
Hemp	leaves	n.a.	19.0–23.0 *	n.a.	n.a.	8.1–18.6 *	[100]
	stem	n.a.	12.8–19.6 *	n.a.	n.a.	0.5–1.6	
	root	n.a.	6.8–7.4 *	n.a.	n.a.	0.80 *	
Herbs de Provence	mixture of herbs	n.a.	n.a.	n.a.	n.a.	3.13–3.27	[19]
Horsetail	loose herbs	n.a.	n.a.	n.a.	n.a.	1.91–2.63	[99]
Marjoram	herbs	n.a.	n.a.	n.a.	n.a.	3.70–3.86	[19]
Mint	leaves	n.a.	n.a.	n.a.	n.a.	2.70–3.34	
Nettle	loose herbs	n.a.	n.a.	n.a.	n.a.	2.33–3.09	[99]
Oregano	leaves	n.a.	n.a.	n.a.	n.a.	2.18–2.72	[19]
Parsley	leaves	n.a.	n.a.	n.a.	n.a.	0.67–0.85	
Peppermint	loose herbs	n.a.	n.a.	n.a.	n.a.	3.36–4.28	[99]
Reumaflos tea	leaves and sachets	n.a.	n.a.	n.a.	n.a.	1.73–2.43	
Rosemary	leaves	n.a.	n.a.	n.a.	n.a.	1.17–1.25	[19]
Reumatfix	sachets	n.a.	n.a.	n.a.	n.a.	1.43–1.89	[99]
Sage	leaves	n.a.	n.a.	n.a.	n.a.	1.24–1.32	[19]
Savory	herbs	n.a.	n.a.	n.a.	n.a.	1.14–1.42	
Spirulina		~75.0	~0.015	<LOD	~0.09	~0.10	[101]
Tarragon	leaves	n.a.	n.a.	n.a.	n.a.	1.00–1.08	[19]
Thyme	leaves and branches	n.a.	n.a.	n.a.	n.a.	7.87–9.70	
Turmeric	rhizoma	n.a.	n.a.	n.a.	n.a.	1.45–1.51	
Willow	bark and loose herbs	n.a.	n.a.	n.a.	n.a.	0.21–0.31	[99]

* depending on the growth stage (analysis conducted after 30, 80, and 90 days of growth); ** *Radix Harpagophytum, Devil’s Claw*; n.a.—data are not available, the analyte was not included in the target list of the applied analytical methods; <LOD—not detected. AA—anthranilic acid; CA—cinnabarinic acid; 3HAA—3-hydroxyanthranilic acid; 3HKyn—3-hydroxykynurenine; KYN—kynurenine; KYNA—kynurenic acid; PIC—picolinic acid; QA—quinolinic acid; TRP—tryptophan; and XA—xanthurenic acid.

**Table 3:** Comparison of concentration of TRP and KP metabolites in fruits and vegetables. 3HKyn, XA, CA, PIC, and QA were not included in a target list of the analytical methods utilized for quantitative analysis.

Fruit/ Vegetable	Form	Concentration Range (mg/kg)					Ref.
		TRP	KYN	3HAA	AA	KYNA	
Apple	–	n.a.	n.a.	n.a.	n.a.	0.002	[38]
Broccoli	–	n.a.	n.a.	n.a.	n.a.	0.41	
Carrot	–	n.a.	n.a.	n.a.	n.a.	0.009	
Cauliflower	–	n.a.	n.a.	n.a.	n.a.	0.047	
Corn	–	n.a.	n.a.	n.a.	n.a.	0.016	
Cucumber	–	n.a.	n.a.	n.a.	n.a.	0.004	
Garlic	–	n.a.	n.a.	n.a.	n.a.	0.027	
Onion	–	n.a.	n.a.	n.a.	n.a.	0.023	
Soybean	roasted	~80.0	~0.13	<LOD	~0.04	~0.01	[101]
Sesame	–	~20.0	~0.05	~0.01	~0.01	~0.02	
Tomato	–	n.a.	n.a.	n.a.	n.a.	0.006	[38]
Pea	–	n.a.	n.a.	n.a.	n.a.	0.009	
Potato	tubers	n.a.	n.a.	n.a.	n.a.	0.24–3.24 *	[17]
	tubers	n.a.	n.a.	n.a.	n.a.	0.13	[38]
	French fries	n.a.	n.a.	n.a.	n.a.	0.1–0.65 *	[17]
	crisps	n.a.	n.a.	n.a.	n.a.	0.03–0.58 *	
	flour	n.a.	n.a.	n.a.	n.a.	0.008–0.04 *	
Pumpkin	–	~80.0	~0.08	~0.01	~0.02	~0.05	[101]
Red paprika	–	n.a.	n.a.	n.a.	n.a.	0.001	[38]
Rice	–	n.a.	n.a.	n.a.	n.a.	0.006	

* concentration in dry weight; n.a.—data are not available, the analyte was not included in the target list of the applied analytical methods; and <LOD—not detected. AA—anthranilic acid; CA—cinnabarinic acid; 3HAA—3-hydroxyanthranilic acid; 3HKyn—3-hydroxykynurenine; KYN—kynurenine; KYNA—kynurenic acid; PIC—picolinic acid; QA—quinolinic acid; TRP—tryptophan; and XA—xanthurenic acid.

**Table 4:** The content of TRP and its KP metabolites in honey and honeybee products. 3HKyn, 3HAA, AA, QA, PIC, and CA were not included in a target list of the analytical methods utilized for quantitative analysis.

Type of Honey Product	N	Concentration Range (mg/kg)				Ref.
		TRP	KYN	KYNA	XA	
Acacia honey dew	5	0.69–0.78	0.30–0.36	0.11–0.15	0.05–0.28	[15]
Bee pollen	2	n.a	n.a.	0.65	n.a.	[38]
Blackberry	5	0.28–0.35	0.18–0.24	11.65–12.35	<LOD–0.02	[15]
Buckwheat	2	n.a.	n.a.	0.33	n.a.	[14]
	2	n.a.	n.a.	0.18	n.a.	[38]
Chestnut	5	0.07–0.12	0.10–0.15	103.50–141.15	0.23–0.34	[15]
	5	n.a.	n.a.	129–601	n.a.	[14]
Clover	1	n.a.	n.a.	0.34–0.75	n.a.	
Eucalyptus	5	1.57–1.78	2.14–4.47	0.49–0.54	0.03–0.60	[15]
	1	n.a.	n.a.	11.30	n.a.	[14]
Fir	1	n.a.	n.a.	1.06	n.a.	
Forest	5	9.56–9.94	0.14–0.18	1.15–1.18	<LOD–0.02	[15]
Heather	5	0.03–0.11	0.03–0.09	1.01–1.07	0.01–0.35	
Holm oak	5	1.04–1.26	0.02–0.06	0.45–0.50	<LOD	
Honey made from Pueblo plants	1	n.a.	n.a.	3.46	n.a.	[14]
Honey dew	1	n.a.	n.a.	0.12	n.a.	
Lavandin	5	0.63–0.74	0.04–0.23	0.60–0.71	0.01–0.04	[15]
Lavender	2	n.a.	n.a.	0.15	n.a.	[14]
Lavender (French)	5	2.51–2.87	0.84–0.98	0.20–0.26	0.04–0.28	[15]
Lavender (spike)	5	2.73–6.68	3.50–5.37	0.29–0.37	<LOD–0.02	
Linden	1	n.a.	n.a.	0.18–0.39	n.a.	[14]
	2	n.a.	n.a.	0.18	n.a.	[38]
Luceme	1	n.a.	n.a.	0.10	n.a.	[14]
Multifloral	5	1.86–2.16	4.68–5.47	3.01–3.24	0.02–0.03	[15]
	1	n.a.	n.a.	0.09–0.12	n.a.	[14]
	2	n.a.	n.a.	0.88	n.a.	[38]
Oak	5	1.10–1.35	0.13–0.31	1.01–1.05	0.01–0.03	[15]
Orange	5	0.95–1.10	1.07–1.32	0.02–0.08	0.01–0.10	
	1	n.a.	n.a.	0.27–0.61	n.a.	[14]
Pine	1	n.a.	n.a.	14.20	n.a.	
Propolis	2	n.a.	n.a.	1.62	n.a.	[38]
Rosemary	5	4.88–14.64	0.33–0.89	0.12–1.17	<LOD–0.02	[15]
Sunflower	5	0.45–0.53	3.13–3.77	0.31–0.37	0.05–0.10	
	1	n.a.	n.a.	1.73	n.a.	[14]
Sulla	2	n.a.	n.a.	0.22	n.a.	
Thyme	5	0.14–3.50	2.04–3.51	0.40–2.12	0.02–0.34	[15]
	1	n.a.	n.a.	0.14	n.a.	[14]
Winter savory	5	0.18–1.07	0.14–0.23	0.26–0.32	0.01–0.08	[15]

n.a.—data are not available, the analyte was not included in the target list of the applied analytical methods; <LOD—not detected; and N—number of analyzed samples. AA—anthranilic acid; CA—cinnabarinic acid; 3HAA—3-hydroxyanthranilic acid; 3HKyn—3-hydroxykynurenine; KYN—kynurenine; KYNA—kynurenic acid; PIC—picolinic acid; QA—quinolinic acid; TRP—tryptophan; and XA—xanthurenic acid.

**Table 6:** The contents of TRP and some KP metabolites in different types of alcoholic beverages. 3HAA, AA, and CA were not included in a target list of the analytical methods utilized for quantitative analysis.

Sample	ABV * (%)	N	TRP	KYN	3HKyn	KYNA	XA	QA	PIC	Ref.
			CR (mg/L)	CR (µg/L)	CR (µg/L)	CR (µg/L)	CR (mg/L)	CR (µg/L)	CR (µg/L)	
Beer	0–7.6	6	4.8–31.1	28.7–86.3	n.a.	16.9–52.0	n.a.	n.a.	n.a.	[18]
		19	n.a.	n.a.	n.a.	0.5–5.2	n.a.	n.a.	n.a.	[11]
Cider	2.5–13	2	n.a.	n.a.	n.a.	0.32	n.a.	n.a.	n.a.	
Cognac	40–60	1	n.a.	n.a.	n.a.	0.06	n.a.	n.a.	n.a.	
Liquer	~15	1	n.a.	n.a.	n.a.	0.1	n.a.	n.a.	n.a.	
Mead	10–14	15	n.a.	n.a.	n.a.	9.4–38.1	n.a.	n.a.	n.a.	
Red wine	<14	7	n.a.	n.a.	n.a.	3.3–10.9	n.a.	n.a.	n.a.	
		4	1.0–1.8	<LOQ	n.a.	82.4–179.7	n.a.	n.a.	n.a.	[18]
Vodka	40–50	3	n.a.	n.a.	n.a.	0.07–1.6	n.a.	n.a.	n.a.	[11]
Whisky	40–50	1	n.a.	n.a.	n.a.	0.06	n.a.	n.a.	n.a.	
Wine	<14	7	n.a.	n.a.	n.a.	1.4–4.7	n.a.	n.a.	n.a.	
		4	<LOD–8.0	<LOD–14.8	<LOD	1.2–65.0	n.a	<LOD–307.1	<LOD–70.2	[116]
		-	n.a.	n.a.	n.a.	n.a.	~10.0	n.a.	n.a.	[117]

* alcohol by volume. n.a.—data are not available, the analyte was not included in the target list of the applied analytical method; CR—concentration range; <LOD—not detected; <LOQ—not quantified; and N—number of analyzed samples. AA—anthranilic acid; CA—cinnabarinic acid; 3HAA—3-hydroxyanthranilic acid; 3HKyn—3-hydroxykynurenine; KYN—kynurenine; KYNA—kynurenic acid; PIC—picolinic acid; QA—quinolinic acid; TRP—tryptophan; and XA—xanthurenic acid.

**Table 7:** The contents of TRP and some KP metabolites in different types of tea and coffee. 3HAA, 3HKyn, AA, CA, and XA were not included in a target list of the analytical methods utilized for quantitative analysis.

Tea/Coffee	Brand/Type	Concentration Range					Ref.
		TRP	KYN	KYNA	QA	PIC	
Black tea	Ceylon Kenilworth	n.a.	n.a.	5.25–5.58 µg/mL	n.a.	n.a.	[12]
	Kenya Original GFOP Milima	n.a.	n.a.	4.99–5.57 µg/mL	n.a.	n.a.	
	Assam TGFOP 1 Tezpore and Gogra	n.a.	n.a.	2.95–3.89 µg/mL	n.a.	n.a.	
	Assam TGFOP 1 Tezpore	n.a.	n.a.	2.38–3.92 µg/mL	n.a.	n.a.	
	Pu erh	n.a.	n.a.	1.90–2.23 µg/mL	n.a.	n.a.	
	China Huang Jing Cha	n.a.	n.a.	1.60–2.19 µg/mL	n.a.	n.a.	
	not specified	248.0–310.0 mg/kg a	0.05–0.28 mg/kg a	0.9–3.4 µg/kg a	n.a.	0.1–0.25 mg/kg a	[119]
Grean tea	Ginkaku Sencha	n.a.	n.a.	6.58–10.81 µg/mL	n.a.	n.a.	[12]
	Japan Tamaryokucha	n.a.	n.a.	6.54–8.53 µg/mL	n.a.	n.a.	
	China Green Yunnan	n.a.	n.a.	3.57–3.96 µg/mL	n.a.	n.a.	
	Asairi Houjicha gojobashi	n.a.	n.a.	3.10–4.32 µg/mL	n.a.	n.a.	
	Gunpowder Temple of Heaven	n.a.	n.a.	2.61–3.11 µg/mL	n.a.	n.a.	
	Che Nhai Dac Biet Jasmine	n.a.	n.a.	0.46–0.57 µg/mL	n.a.	n.a.	
	not specified	39.0–140.0 mg/kg a	0.07–0.64 mg/kg a	0.99–3.0 µg/kg a	0.17–0.32 mg/kg a	n.a.	[119]
Herbal tea	Rooibos Origina	n.a.	n.a.	0.11–0.31 µg/mL	n.a.	n.a.	[12]
Oolong tea	not specified	144.0–395.0 mg/kg a	0.09–0.55 mg/kg a	0.80–3.40 µg/kg a	0.17–0.32 mg/kg a	n.a.	[119]
White tea	Xue Long	n.a.	n.a.	2.79–3.01 µg/mL	n.a.	n.a.	[12]
	China Pai Mu Tan	n.a.	n.a.	2.27–2.80 µg/mL	n.a.	n.a.	
Yellow tea	China Huang Da Cha	n.a.	n.a.	2.13–3.67 µg/mL	n.a.	n.a.	
Ground coffee	Tchibo Family	n.a.	n.a.	0.31–0.34 µg/mL	n.a.	n.a.	
	Cafe Prima Finezja	n.a.	n.a.	0.20–0.23 µg/mL	n.a.	n.a.	
	Douwe Egberts crema silk	n.a.	n.a.	0.17–0.20 µg/mL	n.a.	n.a.	
	Tchibo Exclusive 100% Arabica	n.a.	n.a.	0.06–0.11 µg/mL	n.a.	n.a.	
	Jacobs Kronung	n.a.	n.a.	0.08–0.09 µg/mL	n.a.	n.a.	
	MK Café Premium	n.a.	n.a.	0.05–0.06 µg/mL	n.a.	n.a.	
Instant coffee	Tchibo Exclusive100% Arabica	n.a.	n.a.	0.53–0.73 µg/mL	n.a.	n.a.	
	Tchibo Gold selection crema	n.a.	n.a.	0.29–0.34 µg/mL	n.a.	n.a.	
	Tchibo Family Classic	n.a.	n.a.	0.26–0.31 µg/mL	n.a.	n.a.	
	Nescafé Classic	n.a.	n.a.	0.20–0.25 µg/mL	n.a.	n.a.	
	Jacobs Cronat Gold	n.a.	n.a.	0.16–0.22 µg/mL	n.a.	n.a.	
Whole-bean coffee	Costa Rican Royal Tarrazu	n.a.	n.a.	0.03–0.05 µg/mL	n.a.	n.a.	
	Dallmayr Standard 100% Arabica	n.a.	n.a.	0.02–0.03 µg/mL	n.a.	n.a.	

a—estimated based on data collected from different shooting periods; n.a.—data are not available, the analyte was not included in the target list of the applied analytical methods; and N—number of analyzed samples. AA—anthranilic acid; CA—cinnabarinic acid; 3HAA—3-hydroxyanthranilic acid; 3HKyn—3-hydroxykynurenine; KYN—kynurenine; KYNA—kynurenic acid; PIC—picolinic acid; QA—quinolinic acid; TRP—tryptophan; and XA—xanthurenic acid.

## Data Availability

Data sharing not applicable.

## Abbreviations

AA—anthranilic acid; ACMS—2-amino-3-carboxymuconic 6-semialdehyde; ACMSD—2-Amino-3-carboxymuconic acid semialdehyde decarboxylase; AMS—2-amino-3-muconic acid-6-semialdehyde; APCI—atmospheric pressure chemical ionization; BBB—blood–brain barrier; CA—cinnabarinic acid; ESI—electrospray ionization; FAM—N′-formylkynurenineformamidase; 3HAA—3-hydroxyanthranilic acid; 3-HAAO—3-hydroxyanthranilic acid 3,4-dioxygenase; 3HKyn—3-hydroxykynurenine; HPLC-DAD—high-performance liquid chromatography connected with a diode array detector; HPLC-FD—high-performance liquid chromatography connected with a fluorescence detektor; IDO—indoleamine-2,3-dioxygenase; KAT—kynurenine aminotransferase; KMO—kynurenine mono-oxygenase; KP—kynurenine pathway; KYN—kynurenine; KYNA—kynurenic acid; KynA—kynureninase A; KynB—kynureninase B; LC—liquid chromatography; LLE—liquid–liquid extraction; LOD—limit of detection; NFK—N-formylkynurenine; PIC—picolinic acid; QA—quinolinic acid; QPR—quinolinate phosphoribosyl transferase; QuEChERS—quick, easy, cheap, effective, rugged, and safe; ROS—reactive oxygen species; SPE—solid phase extraction; TDO—tryptophan-2,3-dioxygenase; TLRs—Toll-like receptors; TRP—tryptophan; and XA—xanthurenic acid.
